# Detection of Inferior Alveolar Nerve Injury and Prediction of Sensory Recovery

**DOI:** 10.1155/ijod/6657953

**Published:** 2026-07-08

**Authors:** Yabing Dong, Yunbo Hao, Yiwen Wang, Wenbin Wei, Minjie Chen, Chuangqi Yu

**Affiliations:** ^1^ Department of Oral Surgery, Shanghai Ninth People’s Hospital, Shanghai Jiao Tong University School of Medicine, Shanghai, China, shsmu.edu.cn; ^2^ Shanghai Key Laboratory of Stomatology and Shanghai Research Institute of Stomatology, Shanghai, China; ^3^ National Center for Stomatology, National Clinical Research Center for Oral Diseases, Shanghai, China, njucm.edu.cn; ^4^ Department of Stomatology, Shanghai Tenth People’s Hospital, Tongji University, Shanghai, China, tongji.edu.cn

## Abstract

**Objectives:**

The aim of this study was to evaluate the degree of inferior alveolar nerve (IAN) injury by using current perception threshold (CPT) detection, electrical pulp test (EPT), and two‐point discrimination (2PD). This is a retrospective cohort study conducted to explore the prognostic value of the above three detection methods for sensory recovery of IAN injury.

**Materials and Methods:**

A total of 72 patients with unilateral IAN injury following mandibular third molar extraction were included in this retrospective cohort study. The visual analog scale (VAS), CPT, EPT, and 2PD tests were performed on patients, and the values on the healthy and affected sides of the three tests were compared and the correlation with prognosis was analyzed.

**Results:**

During the follow‐up, 56 patients were classified into the recovery group, with an average recovery time of (3.7 ± 1.2) months and 16 patients were set as the non‐recovery group. The differences in CPT, EPT, and 2PD between the healthy and affected sides of all patients were statistically significant (all *p* < 0.05). Among the data of the affected side of patients in recovery group and the non‐recovery group, only the differences at 5 Hz of CPT and EPT were statistically significant (both *p* < 0.05).

**Conclusion:**

CPT, EPT, and 2PD can all evaluate whether there is IAN injury. The values measured at 5 Hz of CPT and posterior teeth EPT can evaluate the prognosis of nerve recovery, and the corresponding exploratory cut‐off values (CPT Δ40, EPT Δ9) have good diagnostic performance for prognostic judgment.

## 1. Introduction

The inferior alveolar nerve (IAN), a branch of the mandibular branch of the third branch of the trigeminal nerve, mainly governs the dental pulp, periodontal ligament, alveolar bone of mandibular teeth, the labial and buccal gingiva of the lower anterior teeth, the lower lip mucosa, skin, the pain, touch, and proprioception of the skin of the chin [1]. Once the IAN is damaged, the sensation in its main governing area will be temporarily or permanently lost. Symptoms such as numbness of the lower lip, chin, and mandibular gingiva on the affected side often occur, and in severe cases, it affects the patient’s speech, diet, and other daily activities [2]. IAN injury is often caused by alveolar surgeries, orthognathic surgeries, and jaw surgeries, among which the extraction of mandibular wisdom teeth is the most prevalent cause. Therefore, when the IAN is damaged, it is extremely important to assess the nerve damage and predict the prognosis of the patient, which provides critical clinical guidance for the clinical treatment of IAN injury. The existing ZUNIGA and ESSICK detection systems are overly cumbersome and highly subjective [3]. Meanwhile, there is no definite method to effectively evaluate the prognosis of patients with nerve injury.

This study intends to adopt simpler, quicker, and relatively objective nerve injury examinations: current perception threshold (CPT) [4–6], electrical pulp test (EPT) [7], and two‐point discrimination (2PD) [8–10] for quantitative assessment of IAN injury and analyze the identification of nerve injury and its correlation with prognosis, with the aim of establishing a detection system for IAN injury and finding a more accurate prediction method for injury prognosis to provide guidance for subsequent surgical intervention.

## 2. Materials and Methods

This study was designed as a retrospective cohort study. The study population consisted of patients with unilateral IAN injury who visited our outpatient clinic, and the prognostic value of CPT, EPT, and 2PD for sensory recovery was explored by comparing the differences in test values between the healthy and affected sides, as well as between the recovery and non‐recovery groups.

### 2.1. Research Subjects

Patients who visited the oral surgery outpatient department of the Ninth People’s Hospital Affiliated to the Shanghai Jiao Tong University School of Medicine from July 2018 to January 2022 were included. This study was approved by the Institutional Review Board (IRB) of Shanghai Ninth People’s Hospital Affiliated to Shanghai Jiao Tong University School of Medicine, approval number: (SH9H‐2020‐T188‐2). Written informed consent was obtained from all patients prior to enrollment.

All examinations were performed by a single senior clinician with standardized clinical experience, and the interexaminer reliability was tested (Cronbach’s *α* = 0.89), ensuring consistency of detection standards. The examiner was blinded to patients’ recovery status during sensory testing to minimize assessment bias: examiners were unaware of the patients’ surgical records, injury severity, and recovery expectations to avoid subjective bias in the process of detection and result judgment.

Inclusion criteria: 1. patients with unilateral numbness of the lower lip caused by mandibular third molar extraction. 2. At least 1 month after injury. 3. Patients can understand and cooperate with various examination methods. Exclusion criteria: 1. patients with mental disorders such as anxiety and depression. 2. Tumors or osteomyelitis in the mandible. 3. History of diabetes. 4. Bilateral IAN injury. 5. Those unable to undergo the examination for other reasons.

This study is an exploratory study to screen potential prognostic indicators of IAN injury, and thus, the sample size was not precalculated. The sample size was determined based on the number of eligible patients enrolled in the hospital during the study period. Termination criteria of the study: all patients were followed up regularly every 3 months to record the recovery of numbness. During each follow‐up visit, visual analog scale (VAS) scoring was performed to assess the degree of numbness, and CPT, EPT, and 2PD tests were reconducted if necessary. When the VAS of the patient’s numbness was <3 (1–10), combined with the absence of obvious subjective sensory disturbance described by the patient, it was determined that the patient’s lower lip sensation recovered, and the follow‐up was stopped; when the patient’s numbness persisted for more than 1 year with VAS ≥3, it was determined that the patient’s lower lip sensation did not recover, and the follow‐up was stopped. VAS was used as the simple and rapid assessment criterion in this study due to its high clinical practicability and wide application in neurosensory disturbance evaluation [11, 12], while objective neurosensory tests (CPT, EPT, 2PD, and pin‐prick test) were used as an auxiliary reference for clinical judgment.

### 2.2. Research Methods

#### 2.2.1. Data Collection and Grouping

The general conditions of the enrolled patients, including age, gender, IAN injury time, and other information were collected. A VAS score was performed for the numbness of the patient’s lower lip. Based on the VAS value, the patients were divided into the recovery group (VAS < 3 within 1 year) and the non‐recovery group (VAS ≥3 within 1 year).

#### 2.2.2. Materials and Instruments

Conductive paste (Parker Signa Gel, Medtronic, USA); CPT detector (Neurometer, Neurotron, USA); Digital EPT tester (YS‐DT‐A, Yunsheng Company, China); and Vernier caliper (Model 111–102, Dongguan Sanliang Precision Measuring Instrument Co., Ltd., China) were used.

#### 2.2.3. Detection Methods

##### 2.2.3.1. CPT

The CPT detector was used to test the healthy and affected sides of the patient’s lower lip, respectively. Before the test, the skin of the lower lip was wiped with an alcohol cotton ball. After the disinfection area was dried, two electrodes and conductive paste were attached to the numb area of the patient; the CPT instrument was turned on, the fully automatic CPT test was selected in the program, and the patient was asked to perform tests at three frequencies (2000 Hz, 250 Hz, and 5 Hz), respectively, as prompted by the machine. After each test, the test values at the 3 frequencies were recorded, respectively, and they were input into the data analysis software of the CPT detector for analysis.

##### 2.2.3.2. EPT

The digital EPT tester was used to test the EPT of the healthy and affected posterior teeth of the patient (International Dental Federation [Fédération Dentaire Internationale, FDI] tooth position notation: 34, 35, 36, 37, 44, 45, 46, and 47). After strictly separating and drying the tooth surface with a cotton roll, the tip of the instrument was dipped in a small amount of normal saline,it was placed on the surface of the middle 1/3 of the tooth to be tested, and the on/off key was pressed. The patients were asked to raise their hand when they felt pain or discomfort, then the test of this tooth wasended and the corresponding value was recorded. The average of three times was taken as the final test value.

##### 2.2.3.3. 2PD

The tip of the vernier caliper was used to contact the skin surface of the patient’s chin, with the pressure being slightly depressed on the skin. The patient was asked to indicate whether they feel one point or two points of contact. If the patient was unsure about the number of contact points, it was considered as one point. Measured the healthy and affected sides of the patient, respectively, and repeated twice continuously on each side. If the difference was within 1 mm for two times, the test was ended and the minimum value of the two was taken; if it exceeded 1 mm, multiple tests were performed until the difference was within 1 mm for two consecutive times and the minimum value of the two was taken.

### 2.3. Statistical Methods

Excel 2019 and GraphPad Prism 8.0.1 software were used for statistical analysis. Qualitative data were expressed as *n* (%). Quantitative data that conformed to the normal distribution were expressed as *x* ± *s*. The ages of the two groups were compared using the independent sample *t*‐test; the Mann–Whitney *U* test was used to compare the CPT, EPT, and 2PD results of the healthy and affected sides of the patients and the recovery and non‐recovery groups, respectively. Subsequently, a receiver operator characteristic curve (ROC curve) model was established. By analyzing this curve, the sensitivity, specificity, positive predictive value (PPV), and negative predictive value (NPV) of this test for the prognosis assessment of the nerve were calculated. Finally, consistency analysis was performed on the prediction of nerve recovery by CPT, EPT, and 2PD in patients. *p* < 0.05 indicated that the difference was statistically significant.

## 3. Results

A total of 72 patients were included in this study, including 26 males and 46 females; the average age of the patients was 29.2 (15–43) years. All patients had unilateral numbness of the lower lip and chin, accompanied by discomfort in the lower teeth on the affected side. Among them, 32 patients had left IAN damage and 40 patients had right IAN damage. The average time for all patients to receive nerve tests was 3.5 ± 1.8 months after the operation, with the detection time ranging from 1 to 12 months. There was no statistically significant difference in the postoperative detection time between the recovery group (3.4 ± 1.7 months) and the non‐recovery group (3.7 ± 2.0 months) (*p* > 0.05). Among them, 56 patients had a VAS < 3 during the follow‐up period and were set as the recovery group, with an average recovery time of (3.7 ± 1.2) months. The remaining 16 patients were set as the non‐recovery group. There was no statistically significant difference in age, gender, and injury site between the two groups (Table 1).

**Table 1:** Comparison of general conditions of patients in two groups (*n* = 72).

Item	Recovery group (*n* = 56)	Non‐recovery group (*n* = 16)	*p*‐Value
Age	29.1 ± 5.7	29.6 ± 6.1	0.63
Gender	—	—	0.65
Male	21	5	—
Female	35	11	—
Injury site	—	—	0.23
Left	27	5	—
Right	29	11	—
Postoperative detection time (months)	3.4 ± 1.7	3.7 ± 2.0	0.69

### 3.1. Evaluation and Prognosis Prediction of IAN Injury by CPT

In the CPT tests at the three frequencies of 2000, 250, and 5 Hz, the CPT values of the affected side and the healthy side of the patients in the recovery group were (213 ± 17) and (104 ± 8), (92 ± 10) and (29 ± 6), and (45 ± 7) and (13 ± 3), respectively. The differences in the values of the affected side and the healthy side of the patients in the recovery group in the three‐frequency tests were statistically significant (all *p* < 0.05).

**Figure 1:**
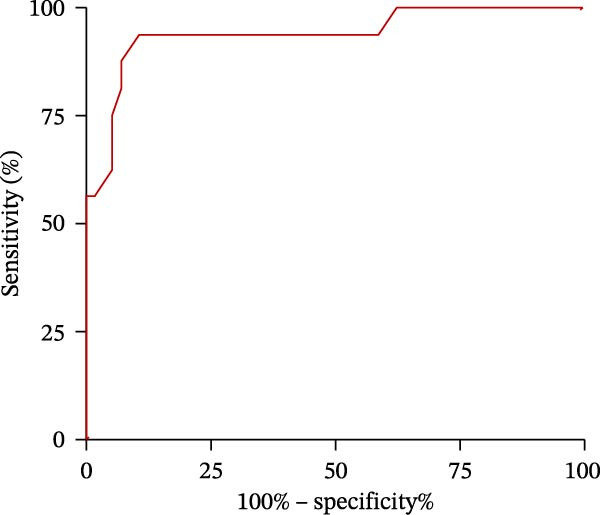
ROC curve for predicting sensory recovery of the lower lip based on the difference between the CPT (5 Hz) of the affected side and the healthy side (AUC = 0.939, 95% CI = 0.86–1.00, and cut‐off = 40).

Comparing the CPT values of patients in the recovery group and the non‐recovery group, the differences on the healthy side of the two groups at 2000, 250, and 5 Hz were not statistically significant; the differences on the affected side at 2000 and 250 Hz were also not statistically significant, but at 5 Hz, the CPT value of the patients in the recovery group was (45 ± 7), and that of the patients in the non‐recovery group was (65 ± 10), and the difference was statistically significant (*p*  < 0.05). An ROC curve model was established based on the difference between the affected side and the healthy side at CPT 5 Hz to predict the sensory recovery situation (Figure 1). Among them, the area under the curve (AUC) was 0.939, and the 95% confidence interval (CI) was 0.86–1.00, *p* < 0.05. This model uses the difference between the affected side and the healthy side, CPT = 40, as the cut‐off value to predict the sensory recovery of the lower lip. Taking the difference of 40 at 5 Hz of CPT as the boundary, among all patients, its sensitivity was 92.86%, its specificity was 87.50%, and the Youden index was 0.80. For patients with a CPT difference ≤ 40, the nerve function of 52 patients recovered and two patients did not recover, and the PPV was 96.30%; for patients with a difference >40, 14 patients did not recover and four patients recovered, and the NPV was 77.78% (Table 2).

**Table 2:** Diagnostic performance metrics of CPT 5 Hz Δ40 for predicting IAN sensory recovery (*n* = 72).

Indicator	Reported value	95% CI
Sensitivity	92.86%	83.7%–97.9%
Specificity	87.50%	61.7%–98.4%
Positive predictive value (PPV)	96.30%	87.9%–99.5%
Negative predictive value (NPV)	77.78%	52.4%–93.6%
Youden index	0.80	0.63–0.98
Area under curve (AUC)	0.939	0.86–1.00

To further explore the independent prognostic value of CPT 5 Hz Δ40 for IAN sensory recovery and eliminate the influence of confounding factors, a binary logistic regression model was established. The dependent variable was sensory recovery (recovery = 1 and non‐recovery = 0), and the independent variable was CPT 5 Hz Δ40 (dichotomized as ≤40 vs. >40). The covariates included were age (continuous variable), gender (dichotomized as female vs. male), injury side (dichotomized as right vs. left), and postoperative detection time (continuous variable). The variables were included in the model based on the following rationales: 1. age and gender are common demographic confounding factors in clinical observational studies, 2. injury side (left/right) may affect the degree of IAN injury and recovery, and 3. postoperative detection time is a key factor affecting neurosensory test results due to the dynamic healing of peripheral nerves. Multivariable regression analysis was performed with sensory recovery as the dependent variable and CPT 5 Hz Δ40 as the independent variable, with age, gender, injury site, and postoperative detection time as covariates (to eliminate the influence of timing variability on prognostic performance). The results confirmed that CPT 5 Hz Δ40 was an independent prognostic factor for IAN sensory recovery after adjusting for postoperative detection time and other confounding factors (*p* < 0.001), while other confounding factors had no significant effect on the prognosis (all *p* > 0.05). The detailed results of the multivariable regression model are shown in Table 3.

**Table 3:** Binary logistic regression analysis for factors associated with IAN sensory recovery (dependent variable: sensory recovery, *n* = 72).

Variable	*β*‐value	Odds ratio (OR)	95% CI	*p*‐Value
CPT 5 Hz Δ40 (≤40 vs. >40)	−4.215	0.015	0.002–0.128	<0.001
Age (years)	0.012	1.012	0.953–1.075	0.716
Gender (female vs. male)	0.208	1.231	0.324–4.679	0.768
Injury site (right vs. left)	0.587	1.806	0.459–7.103	0.395
Postoperative detection time (months)	0.054	1.055	0.829–1.343	0.689

*Note*: *R* ^2^ = 0.582, Hosmer–Lemeshow test *p* = 0.735.

### 3.2. Evaluation and Prognosis Prediction of IAN Injury by the Average Value of Posterior Teeth EPT

In the EPT test, the average value of the affected side of all patients was (18 ± 6), and that of the healthy side was (12 ± 2), and the difference was statistically significant (*p* < 0.05). The comparison of EPT between the healthy side and the affected side of patients in the recovery group and the non‐recovery group is shown in Table 4.

**Table 4:** Comparison of the average EPT of posterior teeth in each group (*n* = 72).

Side of nerve measurement	Recovery group (*n* = 56)	Non‐recovery group (*n* = 16)	*p*‐Value
Uninjured side	11.8 ± 2.0	12.4 ± 2.0	0.72
Injured side	15.8 ± 2.9	26.7 ± 3.9	<0.05

There was no statistically significant difference between the healthy sides of the two groups. While the difference between the affected sides was statistically significant (*p*  < 0.05). When using EPT to evaluate IAN, we took the average EPT difference of 9 between the posterior teeth on the healthy and affected sides as the exploratory cut‐off value. This threshold was established based on the ROC curve analysis of the current study cohort, and external validation in different populations was required to confirm its clinical applicability. Among all patients, the EPT sensitivity was 89.30%, the specificity was 87.50%, and the Youden index was 0.84. For patients with an average EPT difference of posterior teeth ≤9, the nerve function of 50 patients recovered and two patients did not recover, and the PPV was 96.15%; for patients with a difference >9, 14 patients did not recover and six patients recovered, and the NPV was 70.00% (Table 5).

**Table 5:** Diagnostic performance metrics of EPT Δ9 for predicting IAN sensory recovery (*n* = 72).

Indicator	Reported value	95% CI
Sensitivity	89.30%	78.4%–95.9%
Specificity	87.50%	61.7%–98.4%
Positive predictive value (PPV)	96.15%	87.4%–99.5%
Negative predictive value (NPV)	70.00%	45.7%–88.1%
Youden index	0.84	0.66–1.00

### 3.3. Evaluation and Prognosis Prediction of IAN Injury by 2PD

In the 2PD test, the value of the affected side of all patients was (10.9 ± 1.7) mm, and that of the healthy side was (9.8 ± 1.2) mm, and the difference was statistically significant (*p* < 0.05). As shown in Table 6, there was no statistically significant difference between the affected side and the healthy side of patients in the recovery group and the non‐recovery group (*p* > 0.05).

**Table 6:** Comparison of 2PD values of patients in each group.

Side of nerve measurement	Recovery group (*n* = 56)	Non‐recovery group (*n* = 16)	p‐Value
Uninjured side (mm)	9.8 ± 1.3	9.7 ± 0.85	0.70
Injured side (mm)	10.8 ± 1.8	11.2 ± 1.5	0.93

## 4. Discussion

IAN injury is a frequent complication after mandibular third molar extraction, causing persistent numbness and affecting patients’ quality of life [13]. Preoperative imaging such as panoramic radiography and CBCT can help assess anatomical risk [14], but IAN injury may still occur due to intraoperative manipulation, root displacement, or compression [2]. Iatrogenic factors, including insufficient surgical experience and inappropriate techniques, further increase the risk of nerve injury. Therefore, objective and convenient tools for evaluating injury severity and predicting prognosis are urgently needed in clinical practice [13].

The VAS is widely used to assess subjective numbness but is inherently affected by individual perception. In this study, a VAS <3 within 1 year was used as the primary criterion for sensory recovery, consistent with common clinical standards. However, relying solely on VAS may not fully reflect objective neurosensory function. Thus, objective tests, including CPT, EPT, and 2PD, were used as auxiliary references to improve assessment reliability.

Among the three objective methods, 2PD effectively identified IAN injury but showed no significant prognostic value. This can be explained by neurophysiological differences: 2PD mainly evaluates large myelinated A*α* fibers, which are responsible for spatial discrimination [10]. While IAN injury primarily affects small‐diameter A*δ* and C fibers related to pain and numbness. A*α* fibers also possess stronger regenerative capacity, leading to insignificant differences between recovery and non‐recovery groups [12, 15].

CPT enables quantitative, frequency‐specific assessment of Aβ, A*δ*, and C fibers, making it more objective than conventional tests [4, 16]. The larger the CPT value, the higher the degree of nerve injury. In maxillofacial detection, CPT has been used for IAN injury assessment after orthognathic surgery, such as sagittal split osteotomy and other surgical methods. However, there are currently not enough literature to analyze the normal values of IAN and abnormal values after nerve injury. A recent systematic review and meta‐analysis by Vinci et al. [17] comprehensively analyzed neurosensory disturbances following IAN relocation and implant placement and emphasized that the current clinical practice lacks standardized, quantitative tools for neurosensory assessment and prognosis of IAN injury, especially in implant surgery involving direct manipulation of IAN. All three frequencies of CPT can accurately describe the damage of nerve fibers: the values of the healthy and affected sides of the patients in the recovery group at CPT 2000, 250, and 5 Hz were statistically significant (all *p*  < 0.05). Importantly, our study confirmed that only CPT 5 Hz showed significant differences between the recovery and non‐recovery groups.

Based on the difference in CPT at 5 Hz between the affected side and the healthy side, we established a ROC curve model to predict sensory recovery (Figure 1). Through model analysis and calculation, the cut‐off value for predicting lower lip sensory recovery was determined as a CPT difference of 40 between the affected side and the healthy side (designated Δ40), which was most consistent with clinical research data. The cutoff value Δ40 yielded excellent diagnostic performance, and multivariable regression confirmed CPT 5 Hz Δ40 as an independent prognostic factor after adjusting for age, gender, injury side, and postoperative detection time. These findings support CPT at 5 Hz as a reliable and objective predictor of IAN recovery.

In clinical applications in dentistry, EPT is mostly a test for the vitality state of the dental pulp in most cases [7, 18]. EPT also effectively identified IAN injury and showed a clear prognostic value. In this study, the difference between the mean values of the healthy and affected sides of the patients was statistically significant (*p* < 0.05), indicating that EPT can effectively judge IAN injury; the average EPT value of the affected side was significantly lower in the recovery group than in the non‐recovery group, indicating that it has prognostic value for IAN injury.

When using EPT to evaluate IAN, the cut‐off value Δ9 provided good predictive performance. As a routine dental tool, EPT is convenient and can serve as a practical alternative or complement to CPT. However, EPT has limitations: it requires sufficient intact posterior teeth and may be inaccurate in teeth with root canal treatment or restorations.

In summary, this study has several limitations that need to be acknowledged: first, the sample size was relatively small and the study was single‐centered, which may limit the external validity of the conclusions; second, VAS <3 is used as the main criterion for defining sensory recovery, which is subjective and may affect outcome classification; third, the proposed cut‐off values (CPT Δ40, EPT Δ9) are exploratory and data‐driven, lacking internal and external validation. Fourth, the timing of postoperative neurosensory assessments is heterogeneous, which may introduce a potential bias.

Future research should adopt multicenter designs with larger sample sizes to verify the conclusions, incorporate objective neurosensory criteria (e.g., standardized 2PD thresholds and pin‐prick tests) into the definition of sensory recovery, perform internal validation (e.g., bootstrap and cross‐validation) for the proposed cut‐off values, and standardize the timing of postoperative assessments to reduce heterogeneity. Additionally, further studies are needed to explore the combined application of CPT, EPT, and other objective tests to improve the accuracy of prognostic prediction for IAN injury.

## 5. Conclusions

This study mainly conducted 2PD, CPT, and EPT examinations on patients with unilateral IAN injury and studied their diagnostic value and prognosis for IAN injury. It was found that 2PD, CPT, and EPT can all make a clear diagnosis of IAN injury. The mean values of CPT 5 Hz and posterior teeth EPT have high consistency in the prognosis evaluation of IAN injury, and both can effectively evaluate the prognosis of IAN injury with their respective exploratory cut‐off values (CPT Δ40, EPT Δ9). In special cases, they can complement or replace each other. The limitations of this study include small sample size, single‐center design, subjective VAS as the main criterion for defining sensory recovery, and the exploratory nature of the proposed cut‐off values without external validation; future multicenter studies with larger samples are needed to verify the conclusions, and objective neurosensory criteria will be incorporated to improve the accuracy of outcome classification. The research results can not only provide a reference for the prognostic evaluation of IAN injury after mandibular third molar extraction but also have potential application value for the neurosensory assessment of IAN injury in oral implant surgery.

## Author Contributions

**Yabing Dong**: conceptualization, data curation, formal analysis, investigation, methodology, writing – original draft, writing – review and editing. **Yunbo Hao**: data curation, investigation, methodology, validation. **Yiwen Wang**: data curation, formal analysis, visualization. **Wenbin We** **i:** resources, supervision, validation. **Minjie Chen**: investigation, methodology, project administration, supervision, writing – review and editing. **Chuangqi Yu**: conceptualization, funding acquisition, supervision, writing – review and editing.

## Funding

This work was supported by grants from the National Natural Science Foundation of China (Grant 81870785), the Interdisciplinary Program of Shanghai Jiao Tong University (Grant YG2022QN050), the Shanghai Science and Technology Commission (Grant 21Y11903500), and the Rare Disease Registration Project of the Ninth People’s Hospital Affiliated to Shanghai Jiao Tong University School of Medicine (Grant JYHJB202204).

## Conflicts of Interest

The authors declare no conflicts of interest.

## Data Availability

The data that support the findings of this study are available upon request from the corresponding author. The data are not publicly available due to privacy or ethical restrictions.
